# Characterization of a Novel *Tectivirus* Phage Toil and Its Potential as an Agent for Biolipid Extraction

**DOI:** 10.1038/s41598-018-19455-2

**Published:** 2018-01-18

**Authors:** Jason J. Gill, Baixin Wang, Emily Sestak, Ryland Young, Kung-Hui Chu

**Affiliations:** 1 Department of Animal Science, Texas A&M University, College Station, TX 77843 USA; 2 Zachry Department of Civil Engineering, Texas A&M University, College Station, TX 77843 USA; 3 Center for Phage Technology, Texas A&M University, College Station, TX 77843 USA; 4 Department of Biochemistry and Biophysics, Texas A&M University, College Station, TX 77843 USA

## Abstract

The oleaginous bacterium *Rhodococcus opacus* PD630 is metabolically diverse and can be cultivated on various renewable resources to serve as a sustainable triacylglycerol (TAG) feedstock for biodiesel production. Current methods for TAG extraction are costly, but infection of cultures by lytic bacteriophages (phages) may be a viable approach for achieving release of intracellular lipid from oleaginous bacteria such as *R. opacus*. This study reports the novel tectiviral phage Toil capable of releasing intracellular contents including a fluorescent protein marker and TAGs into the supernatant after phage infection of *R. opacus* PD631, a domesticated derivative of strain PD630. Phage Toil is placed in the *Tectiviridae* by its morphology, the presence of a lipid membrane, its genome architecture and the presence of terminal covalently-linked proteins. Toil is the first tectivirus capable of infecting a member of the *Actinobacteria*. Microscopy shows that infected cells do not undergo sudden lysis but instead maintain their original shape for several hours, with the cellular morphology gradually deteriorating. Approximately 30% of intracellular TAGs could be recovered from the culture supernatants of Toil-infected PD631 cells. Phage Toil has potential to be used as an agent in extraction of TAGs from oleaginous bacterium *R. opacus*. **Importance**: This study reported the first tectivirus (Phage Toil) capable of infecting a member of the *Actinobacteria*. In this study, we showed that Phage Toil can infect oleaginous bacterium *Rhodococcus opacus* to release intracellular contents such as a fluorescent protein marker and TAG lipid granules, which can serve as a starting material for biodiesel production. This study demonstrates a new method to extract TAGs by using this phage. Additionally, Phage Toil can be a new model phage to advance knowledge regarding phage infection mechanisms in *Rhodococcus* and other mycolic acid-containing bacteria such as *Mycobacterium*.

## Introduction

Biodiesel is a promising clean liquid fuel with great potential to supplement and replace fossil-derived diesel, because biodiesel is nontoxic and generates much less greenhouse gas compared to fossil fuels^1^. Biodiesel is also renewable; it can be manufactured using starting material, a natural lipid triacylglycerol (TAG) which can be derived from various lipid feedstocks, including plant seeds, and animal oils and fats, microalgae, and oleaginous yeast and bacteria^2,3^. However, the current production cost of biodiesel is high, due in part to the high costs associated with TAG extraction. TAGs are commonly obtained from oil resources such as microalgae, plants and animals through solvent extraction methods^2,3^. While solvent-based extraction is rapid, the process is not only costly but also requires downstream processes to separate solvent from TAGs. Additional concerns about the solvent-based extraction methods include the use of large amount of costly toxic solvents, the concomitant potential for residual solvent in the final product, and the production of secondary waste stream. Super-critical carbon dioxide extraction has been applied for TAG extraction due to its free of toxic solvent, yet, the process is energy intensive and not as efficient^4^. Therefore, a more economic and sustainable means for TAG extraction is needed.

Among many oleaginous microorganisms, the bacterium *Rhodococcus opacus* PD630 has attracted increasing interest due to its ability to accumulate TAGs up to 76% of its cell dry weight under nitrogen-limited conditions^5^. *R. opacus* is a mycolic acid- producing member of the order *Corynebacteriales*, is metabolically diverse, and has been showed to produce TAGs from a broad range of organic compounds including lignocellulosic biomass^6–13^. Using this model oleaginous bacterial strain to produce TAGs offers a unique opportunity for us to develop a new means for TAG extraction –using bacteriophages to lyse cells for TAG release.

Bacteriophages (phages) are the viruses of bacteria and they can infect bacterial cells, leading to intracellular replication and then lysis of the host. The continued emergence of antibiotic-resistant pathogens and increasing disfavor of chemical biocides has resulted in increased interest in phage as biocontrol agents for foodborne pathogens, or diseases in plants and humans^14,15^. Applications of phage technologies to environmental problems are limited to wastewater treatment plants^16,17^. Recently, our laboratory has successfully demonstrated bioextraction of high-valued biopolymer, polyhydroxybutyrate (PHB), from *Pseudomonas oleovorans* grown on synthetic crude glycerol (with alcohol and high salt content)^18^. Accordingly, *R. opacus* phages, especially phages against strain PD630 would be of great interest because, in principle, they could specifically infect a TAG-accumulating host, leading to release of the intracellular contents, including the TAGs. Here we report isolation and characterization of a novel *R. opacus* phage and demonstrate its ability to be used as bioextraction agent in an environmentally friendly, safe and relatively low-cost approach for lipid extraction.

## Results and Discussion

### Isolation and characterization of phage Toil

Phage Toil was isolated on *R. opacus* strain PD631 from a soil sample collected near an open-air aerobic digestion basin of a local wastewater treatment plant in College Station, TX. Strain PD631 is a domesticated derivative of strain PD630 that was obtained by repeated subculture of strain PD630 on R2A medium. Phage Toil is able to form well-defined plaques on lawns of strain PD631 but not on the parental strain PD630. Aside from its ability to support plaque formation, no significant differences were observed between PD631 and PD630, including cell morphology, growth rate, 16 S sequence (27 F to 1392 R) and lipid accumulation ability. Such strain domestication following extensive culture in laboratory conditions is a known phenomenon observed in other bacteria^19,20^. Phage Toil appears to be virulent, as stable lysogens could not be isolated following exposure of PD631 to the phage and recovery of bacterial survivors.

### Phage Toil Morphology

**Figure 1:**
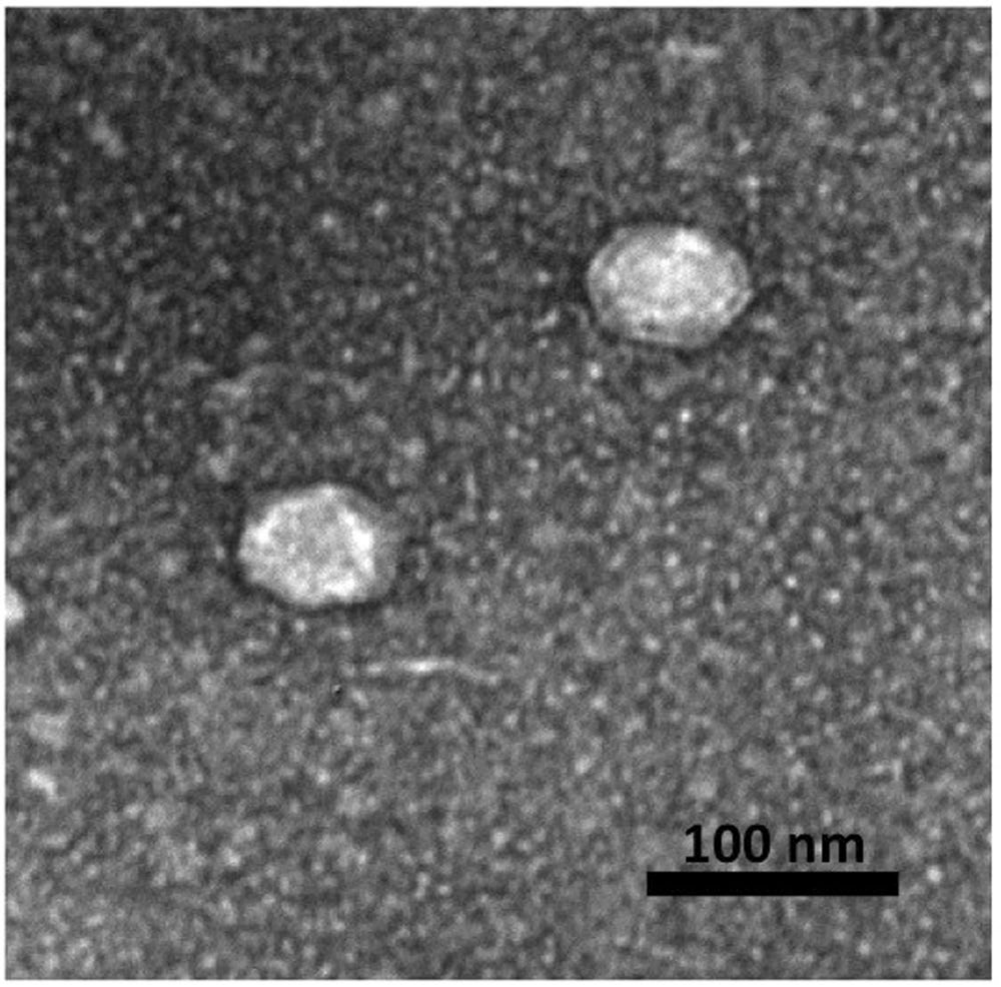
Transmission electron microscopy of phage Toil.

Electron microscopy of phage Toil showed a double-layered phage capsid of an average face-to-face diameter of 54 nm, suggesting the possibility of a lipid membrane associated with the virion (Fig. 1). This possibility was tested by exposing the phage to 15% (v/v) chloroform, upon which a ~95% drop in phage titer was observed after exposure for 30 s at room temperature. The morphology and chloroform-sensitivity of phage Toil suggested that this phage is a novel member of the *Tectiviridae*, of which the type phage is the well-studied enterobacterial phage PRD1.

### Phage Toil Genome

**Figure 2:**
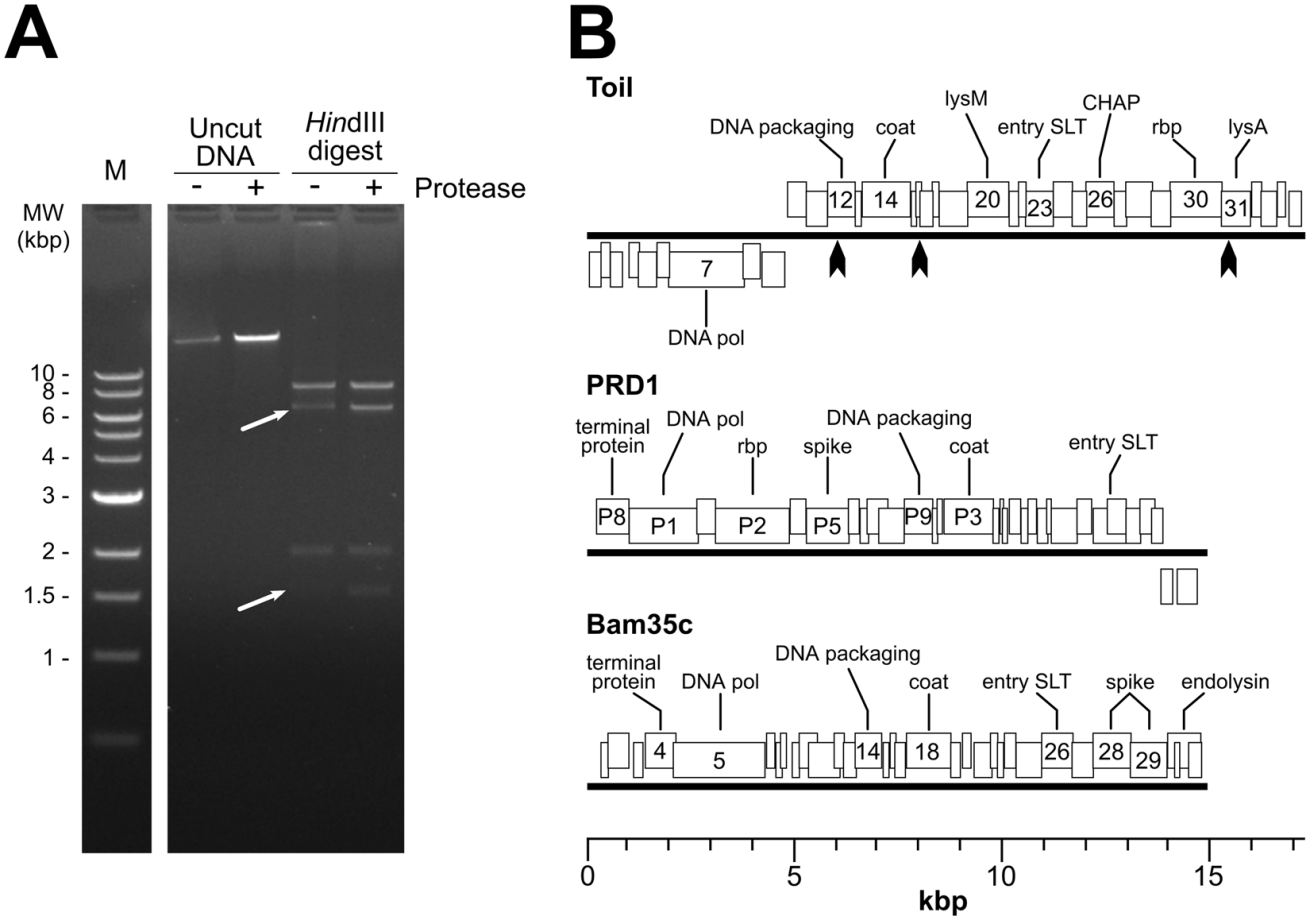
Genome analysis of phage Toil. (**A**) Restriction digest of phage Toil genomic DNA with and without proteinase K treatment. Lane 1, NEB 1 kb molecular weight standard; Lane 2, Untreated DNA; Lane 3, proteinase K treated DNA; Lane 4, HindIII digested DNA; Lane 5, HindIII digested and proteinase treated. White arrows in lane 4 point to restriction fragments from the genomic termini predicted to contain covalently-linked protein; digestion of DNA with proteinase K restores band intensity. Full length gel (Fig. S3) is available in supplementary information. (**B**) Genome maps of phage Toil, enterobacterial phage PRD1 and *Bacillus* phage Bam35c. Heavy black lines represent the DNA molecules, with boxes above the lines representing genes transcribed from the forward strand and boxes below the lines representing genes transcribed from the reverse strand. Predicted functions of major genes are annotated: DNA polymerase (DNA pol), LysM domain-containing protein (lysM), lytic transglycosylase used for phage DNA entry (entry SLT), CHAP domain-containing protein (CHAP), receptor-binding protein (rbp), and predicted endolysin (lysA). ORF numbers or gene names are indicated for genes with annotated functions. Black arrows on the phage Toil map represent the positions of HindIII sites.

The phage Toil genomic DNA (gDNA) was sequenced on an Illumina MiSeq platform, which after assembly generated a single contig at 310-fold coverage. Genome ends were determined by Sanger sequencing directly from the phage gDNA using primers that faced off either end of the assembled contig. The Sanger sequencing signal abruptly stopped or degenerated to noise at discrete nucleotide positions at each end of the genome, which were interpreted as the physical termini of the linear Toil gDNA. The genomes of tectiviral phages such as PRD1 and Bam35c are known to possess covalently linked proteins at their termini. The presence of the terminal proteins affects the migration of phage DNA in gel electrophoresis^21^. As shown in Fig. 2A, Toil gDNA cut with HindIII produces two bands that migrate abnormally; these two bands correspond to the predicted restriction fragments generated from the ends of the phage genome. Digestion of the DNA with proteinase K restores the band migration to the predicted pattern, consistent with the degradation of proteins covalently linked to the gDNA. Due to the presence of the covalently-linked terminal protein, it is possible that a small number of additional bases may remain unsequenced at the termini of the Toil genome.

The gDNA of phage Toil is a non-permuted, linear dsDNA of 17,253 bp encoding 35 predicted proteins (Fig. 2B). The presence of inverted terminal repeats is a feature of tectiviral genomes, as typified by the 110 bp perfect inverted repeats found in phage PRD1^22^. The phage Toil genome is terminated on each end by 72 bp imperfect inverted terminal repeats. These repeats share 75% identity, with 17 mismatches and one gap, similar to the genome of the *B. thuringiensis* tectivirus Bam35c (NC_005258) which also contains imperfect inverted terminal repeats of 74 bp with 80% identity^21^.

Phage Toil was found to be only distantly related to any other phage, including other tectiviruses. With the exception of a short region within the predicted endolysin (see below), there is no detectable DNA similarity between Toil and any organism in the NCBI nr database (E < 1). Only eight of Toil’s 35 predicted proteins have any similarity to other proteins in the database as detectable by BLASTp at E < 0.001, and no Toil protein has directly detectable similarity (BLASTp, E < 10) with any proteins found in other tectivirus genomes. However, as shown in Fig. 2B, the order of the DNA polymerase, packaging ATPase, coat and entry soluble lytic transglycosylase is generally conserved in Toil and other tectiviruses. The left arm of the Toil gDNA containing the DNA polymerase gene is inverted relative to PRD1 and Bam35c. Assuming the DNA polymerase gene marks an early gene cluster whereas the packaging, entry, capsid and lysis proteins are encoded by late genes, the Toil genome has divergent early and late transcriptional units. At 17.2 kb, Toil is also the largest known tectivirus, with phages such as PRD1, Bam35c, AP50, GIL61c and Wip1 having 14–15 kb genomes.

Functional annotation of the Toil genome identified several proteins that provide key tectiviral functions; a complete annotation table is provided in Table S1 in the supplementary information. Besides the aforementioned DNA polymerase, there are genes for the DNA packaging ATPase, the major coat protein, the entry lysozyme, a receptor-binding protein, and the endolysin. Phage Toil gene *7* encodes a single subunit phage DNA polymerase related to the polymerases of other known *Tectiviridae*. In addition to containing a detectable viral/mitochondrial DNA polymerase domain (IPR004868) and palm domain (IPR023211), gp7 also has similarity to the PRD1 DNA polymerase P1 detectable by PSI-BLAST (E = 1 × 10^−64^ at 3 iterations). Toil gene *12* encodes the phage DNA packaging ATPase protein. Gp12 contains a P-loop NTP hydrolase domain (IPR027417) and matches against PRD1 protein P9 with 1 iteration of PSI-BLAST at E = 4 × 10^−10^. PRD1 P9 is required for DNA packaging into the empty capsid and remains associated with the particle following virion maturation^23^.

Toil gene *14* encodes the viral coat protein, based on HHpred matches to the *Sulfolobus* turreted icosahedral virus (STIV) coat protein (2bbd_A, 97.7%) and *Pseudoalteromonas* phage PM2 major capsid protein (2vvf_A, 97.4%). Although these viruses infect different domains of life, their major coat proteins share a common three-dimensional structure, a trimeric double beta-barrel (or “double jelly-roll”) fold; this same fold is also found in adenoviral coat proteins, suggesting the tectiviral and adenoviral coat proteins may share a common ancestor^24,25^. The coat protein of the canonical tectivirus PRD1 also contains this fold, placing phage Toil in the proposed PRD1-adenovirus lineage^26^.

Toil gp30 has similarity detectable by BLASTp to several predicted tail proteins of *R. erythropolis* siphophages including Partridge (AOZ62849, E = 9 × 10^−22^) and Harlequin (AOT23598, E = 3 × 10^−21^). The *R. erythropolis* homologs are approximately twice the length of the 411-residue Toil gp30, and the region of protein sequence identity lies in the C-terminal half of these homologs, which is the region most likely to be involved in receptor-binding activity. Analysis of Toil gp30 by InterProScan finds a C-terminal TNF-like domain (IPR008983), and HHpred detects C1q-like domains at the same site (e.g., 4ous_A, W2nv_A, 99.8%). These domains are known to form trimeric complexes^27^, which is consistent with the trimeric structure of other tectiviral spike proteins^28^. Members of the *Tectiviridae* recognize their host cells via receptor-binding spikes that protrude from each of the virion’s 12 pentameric capsid vertices^29^. The vertex spike of tectivirus PRD1 is composed of three proteins, with the receptor-binding protein P2 anchored to the virion via P5 and P31^28^. Since the TNF and C1q domains are both known to be associated with ligand-binding activity, Toil gp30 most likely functions as the receptor-binding protein.

Toil gp31 contains a centrally-located D-ala-D-ala carboxypeptidase domain (IPR009045) and BLASTp analysis shows similarity to LysA proteins in mycobacteriophages, such as Myrna (YP_002225120, E = 2 × 10^−32^) and Kazan (AMW64361, E = 9 × 10^−10^). In mycobacterial phages of the *Caudovirales* family, LysA is the endolysin responsible for degradation of the host peptidoglycan as part of the terminal lysis event. This activity appears to be conserved in the across viral family lines in the tectivirus Toil. Other mycobacteriophages are known to encode a second lysis protein, LysB, which possesses esterase activity that degrades the outer mycolic acid layer of the cell envelope^30^. No protein with such activity was identified in phage Toil, indicating that this activity is either cryptic, that the phage can accomplish an equivalent disruption of the cell’s outer mycolic acid layer by some other mechanism, or that this activity is not strictly required in this organism. A second lysozyme-like protein in Toil, gp23, is proposed to be a virion-associated lysozyme and not involved in the terminal lysis event. Even though gp23 contains no detectable sequence similarity to any PRD1 protein, it is architecturally similar to the product of PRD1 gene *VII* containing an N-terminal lysozyme-like domain (IPR023346) and a C-terminal transmembrane domain. PRD1 gene *VII* encodes a virion-associated transglycosylase that aids in phage infection, presumably by degrading the cell wall to allow entry of the gDNA into the cell^31^.

The virion morphology, chloroform sensitivity, presence of terminal genomic repeats with covalently-linked terminal proteins, genome size and organization, and the relationship of the predicted Toil major coat protein to the PRD1-like coat proteins of are fully consistent with the placement of Toil within the *Tectiviridae* family of viruses. As of this writing, only 12 tectiviral genomes have been deposited into the public databases (INSDC), falling into two major lineages, represented by enterobacterial phage PRD1 and *Bacillus* phage Bam35c. Based on current viral taxonomic guidelines^32^, phages with less than 95% nucleotide similarity may be considered different species; the lack of any direct DNA or protein similarity (as detectable by BLASTn or BLASTp, respectively) between phage Toil and other tectiviruses marks Toil as the founder of at least a new species within the Tectiviridae, if not a new genus, which may represent a third lineage of tectivirus. At present, the relationship between Toil and other tectiviruses is only detectable at the protein level by more sensitive analyses such as PSI-BLAST or the presence of shared conserved domains. To our knowledge, Toil is the first report of a tectivirus that can infect a member of the *Actinobacteria*. This is also the first example of a virulent tectivirus capable of infecting a Gram-positive host, as the known *Bacillus* Bam35-like phages are temperate and the virulent PRD1 only infects Gram-negative hosts^33,34^.

### Growth Characteristics of Phage Toil

**Figure 3:**
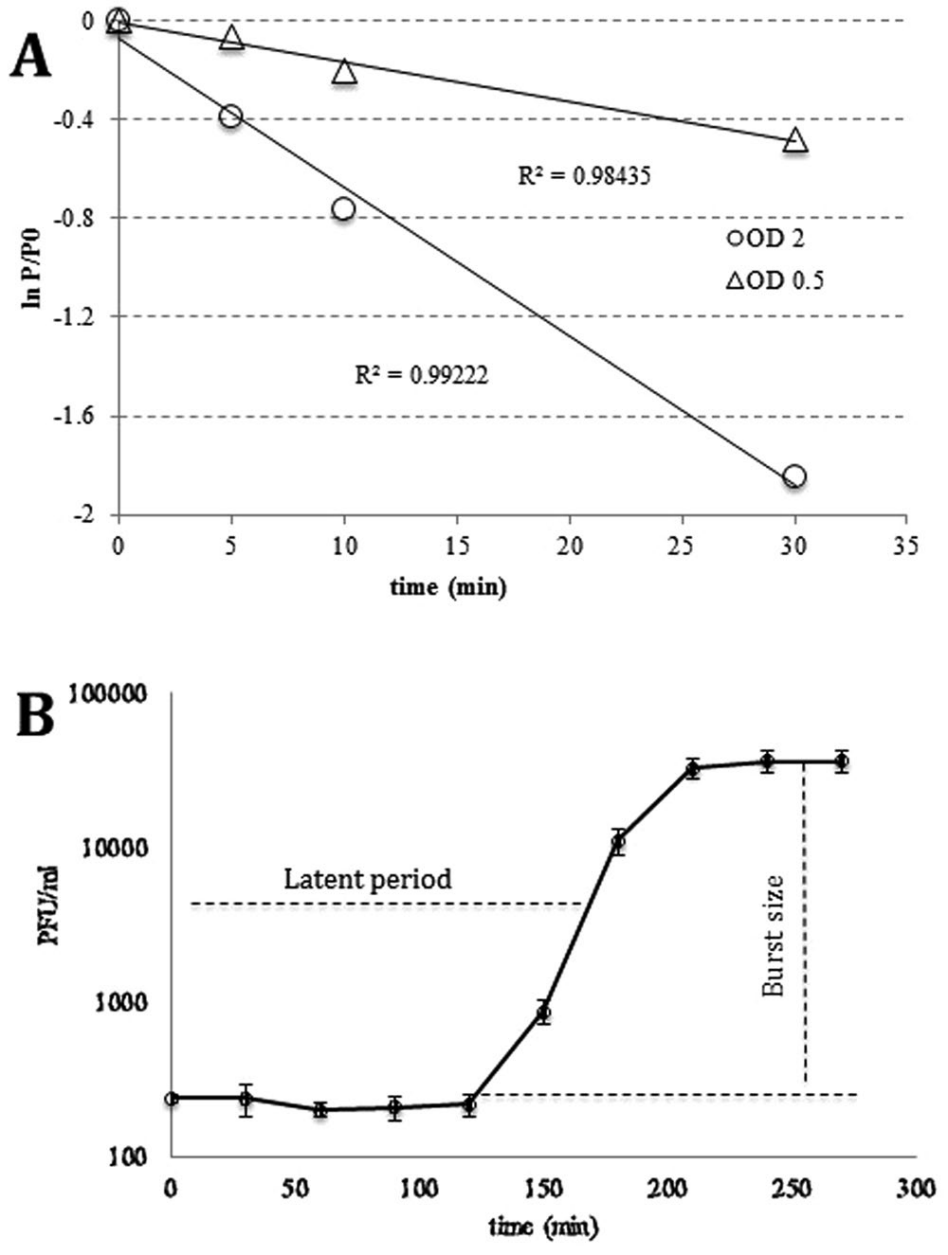
Adsorption and one-step growth experiments of phage Toil. (**A**) Adsorption tests in the presence of 5 mM Ca^2+^. P is the free phage titer, P0 is the free phage titer at time zero. (**B**) One-step growth curve with 5 mM Ca^2+^. Error bars represent triplicate data.

Minimal inhibitory concentration (MIC) experiments showed that phage Toil was able to clear PD631 cultures with a minimal input multiplicity of infection (MOI) of 0.1 (Fig. S1 in supplementary information). The maximal titer of Toil plate lysate achieved was 3 × 10^9^ PFU/ml. To optimize phage adsorption, different concentrations of Ca^2+^ and/or Mg^2+^ ranging from 0 to 10 mM were screened by assessing the titer of unadsorbed phage Toil after 10 min of exposure to host cells (Fig. S2 in supplementary information). The cation Ca^2+^, but not Mg^2+^, was found to affect Toil adsorption, with optimal adsorption occurring at 5 mM Ca^2+^. Divalent cations such as Ca^2+^ and Mg^2+^, are known to affect the ability of phages to adsorb to their hosts^35^. Divalent cations have been shown to play a role in DNA entry, but do not appear to be crucial for adsorption, in tectiviral phage Bam35c which infects *B. thuringiensis*^36^. Under the optimized condition of 5 mM Ca^2+^, the adsorption constant was determined to be 6 × 10^−10^ ml/min (Fig. 3A). One-step growth tests performed with an input MOI of 0.001 showed that phage Toil has a very long latent period (160 min, interpreted as the mid-point of the rise period), generating a burst size of ~23 PFU per infected cell (Fig. 3B).

### Bacteriophage lysis of *R. opacus* with accumulated TAG

**Figure 4:**
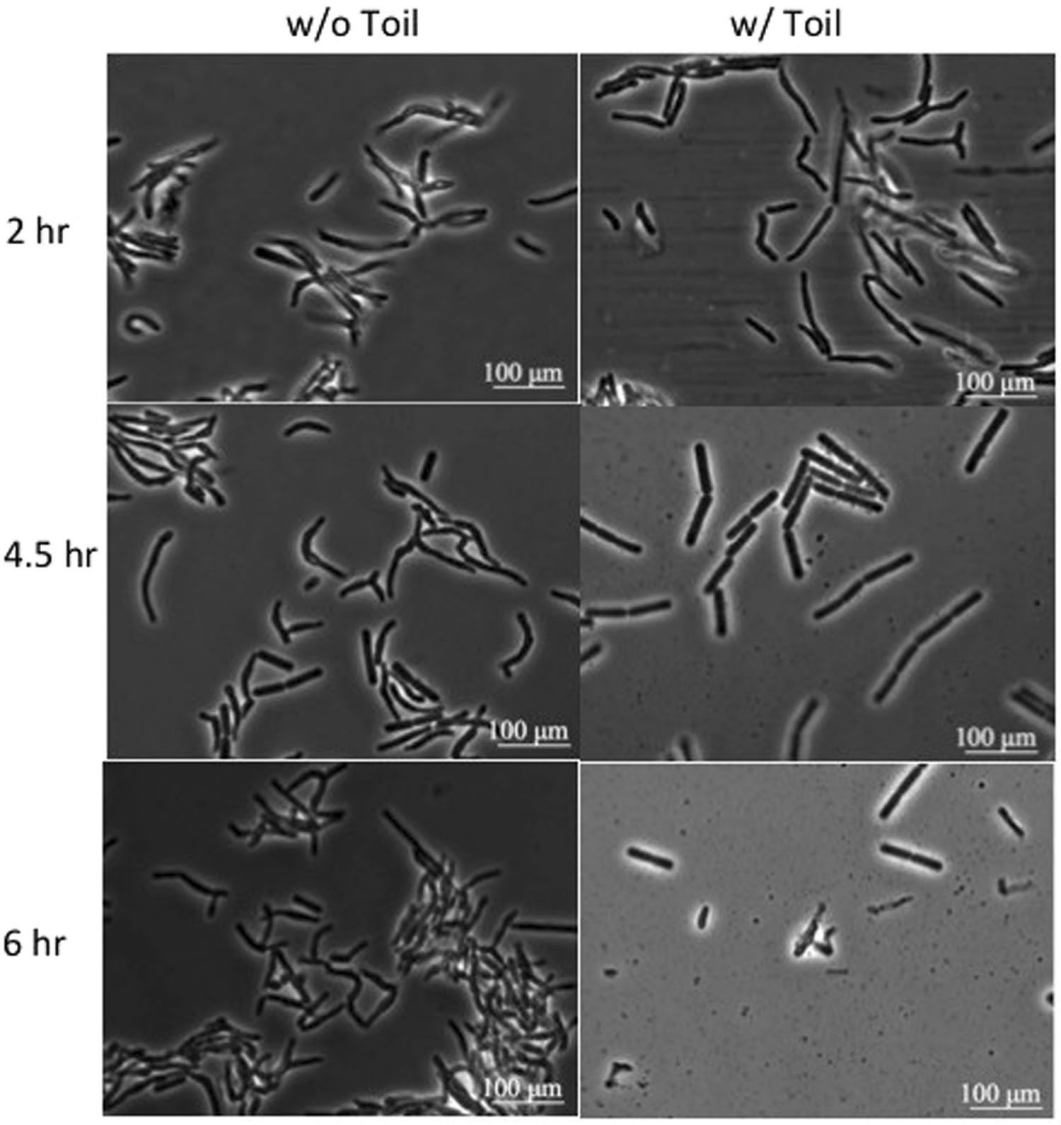
Phase contrast microscopy of the phage Toil infected strain PD631. PD631 was grown after lipid accumulation to OD_600_~15 and then infected as described in Methods.

**Figure 5:**
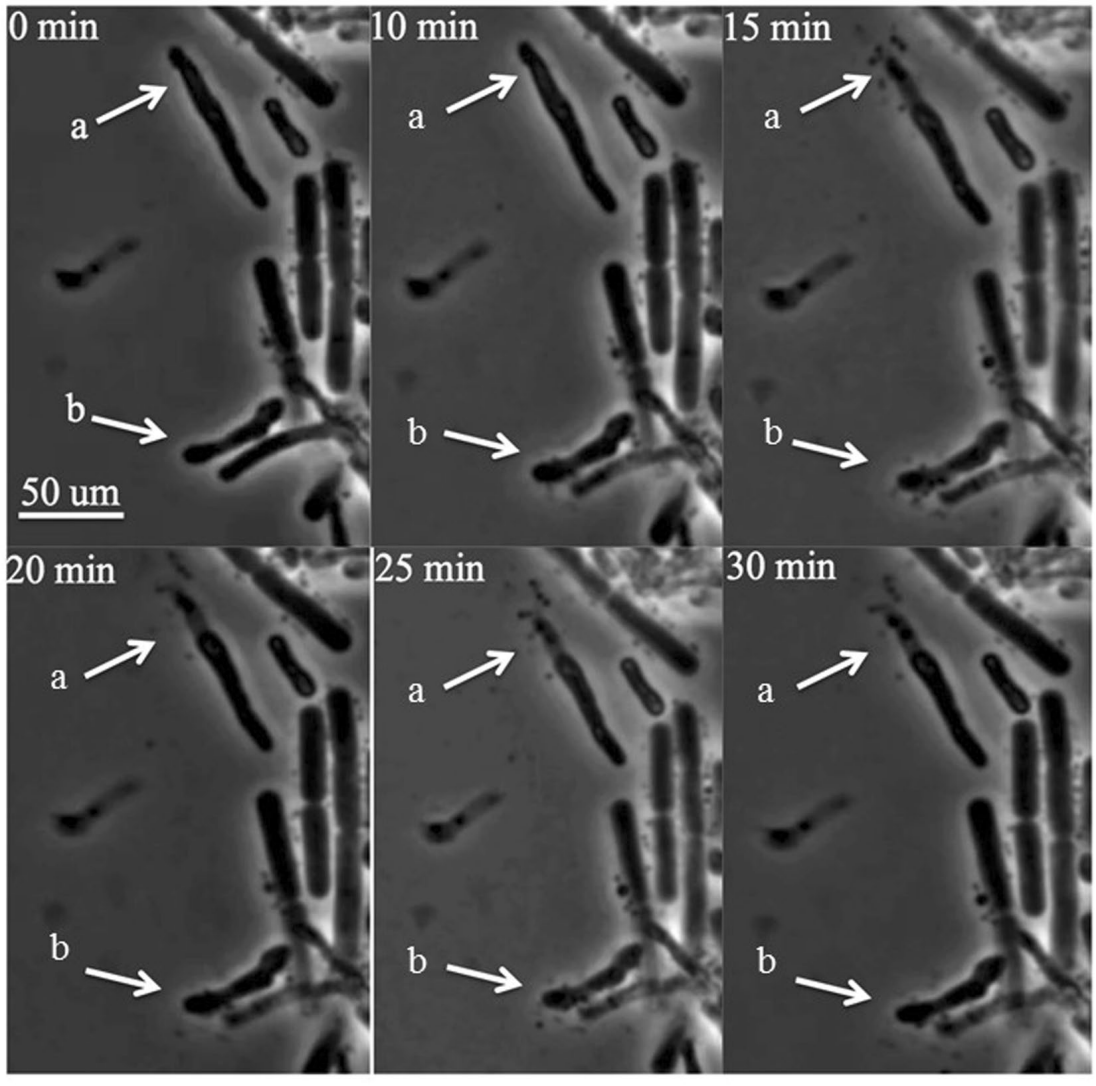
Infection video frames of the phage Toil infecting strain PD631. Video was taken during the period of 3–3.5 hours after PD631 cells induced for lipid accumulation were infected, as described in Methods. Arrows (a) and (b) showed cell lysis over time.

Most bacteriophages can only propagate on growing cells^37^. However, lipid accumulation can only be achieved after PD631 reaches stationary phase, when nitrogen is limiting and carbon is in excess^9^. To overcome this problem, we controlled the growth phase of PD631 by adjusting the nitrogen content in the medium at different time points to allow for lipid accumulation before phage infection. Strain PD631 was first grown in balanced ammonium mineral salts medium (AMS, 10 g/l gluconate and 0.5 g/l NH_4_Cl) to reach stationary phase. After the culture reached stationary phase, the cells were then incubated with mineral salts medium containing no nitrogen but 10 g/l gluconate to accumulate lipid. Upon completion of TAG accumulation, the cells were stimulated to return the active growth state by providing excess nitrogen in the growth medium for phage infection; the TAG-filled culture was resuspended in phage Toil lysate (in R2A broth medium) containing 0.5 g/l additional NH_4_Cl. Due to cell aggregates and debris that introduced significant turbidity, we found that it was not possible to monitor cell lysis by direct measurement of culture optical density. Thus, liquid samples were taken at time intervals for observation under phase contrast microscopy (Fig. 4). The microscopic analysis showed that PD631 underwent lysis by Toil over a broad time interval of 2 to 6 h, consistent with the results observed from the one-step growth tests. The morphological changes of individual PD631 cells during the lysis process were revealed by video micrographs taken during the rise period (Fig. 5).

Although lysing cells underwent abrupt release of the cytoplasmic contents, the overall rod-like morphology was maintained for up to several hours. This is significantly different from the lysis observed with canonical phages of *E. coli*, where lysed cells deteriorate into shapeless debris almost instantly^38^. Presumably this morphological stability is related to the complex composition of the mycolata cell wall, especially since Toil appears to lack a LysB-like esterase for destruction of the covalent links between the outer mycolic acid membrane and the cell wall.

### Release of intracellular contents from PD631 by phage infection

**Figure 6:**
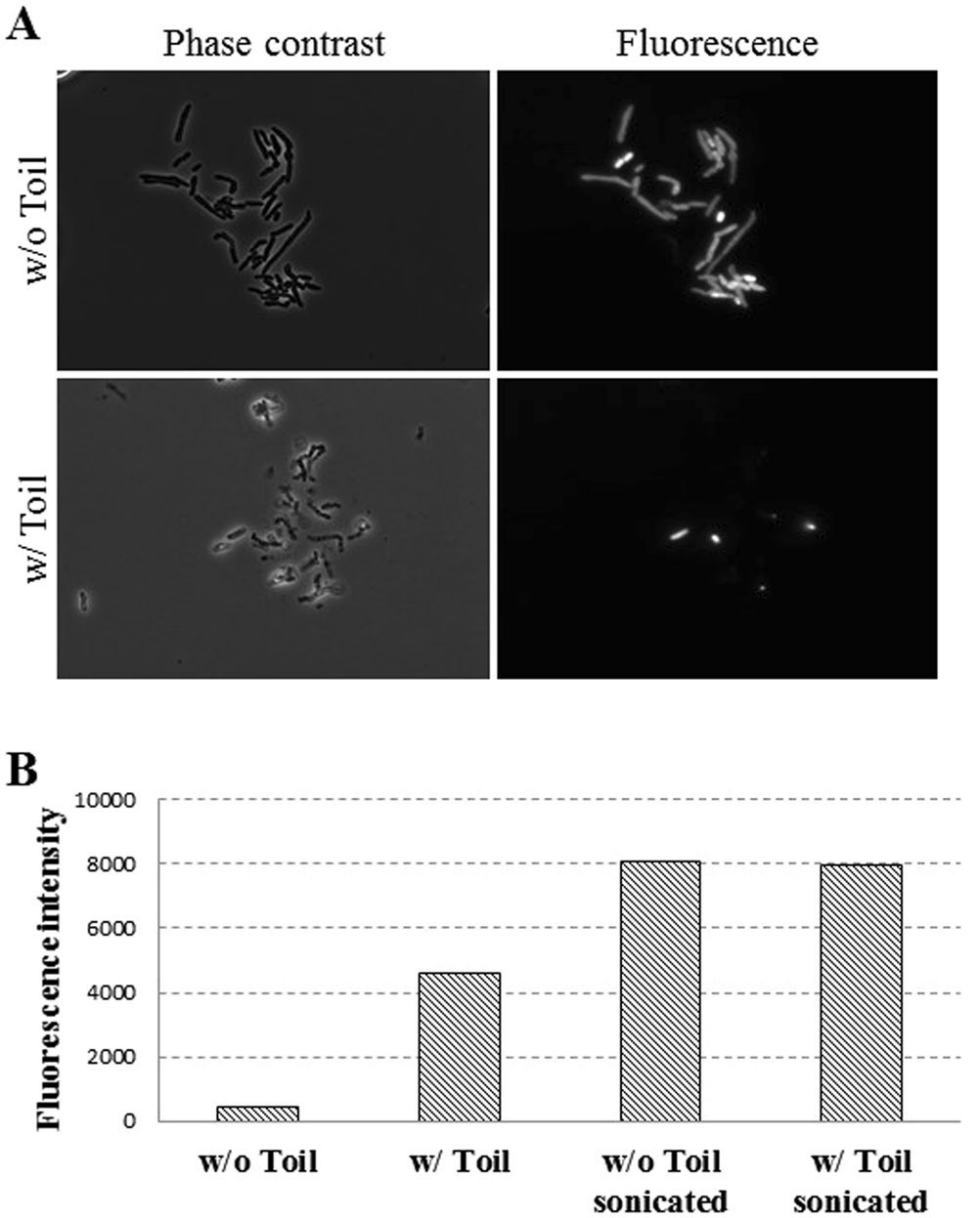
mCherry released from PD631 after phage Toil infection. (**A**) Phase contrast and fluorescence microscopy of mCherry-expressing PD631 cells infected with phage Toil, as described in Methods. Phase contrast and fluorescence images taken at 6 h after infection are shown. Cells without phage are intact under phase contrast and contain mCherry in the cytoplasm; cells exposed to phage are disrupted and most lack mCherry signal, indicating the protein was lost to the medium following lysis. (**B**) Fluorescence intensity in the medium of phage Toil-infected PD631 cells expressing mCherry. At 6 hours post-infection, the culture supernatants from infected and uninfected cells were measured for fluorescent signal, indicating release of intracellular mCherry into the medium. Cells disrupted by sonication provide total release of fluorescent protein, and exposure to phage produces partial release of intracellular protein.

To demonstrate that phage-mediated cell lysis can release intracellular contents from PD631, phage Toil was used to infect PD631 carrying the plasmid pTACHis18/mCherry. After incubation for 6 h with phage, fluorescence microscopy was used to image cells (Fig. 6A). Most of the uninfected cells exhibited diffuse fluorescence, consistent with cytoplasmic accumulation of mCherry, whereas only a few cells from the infected culture exhibited fluorescence, and those that did retained phase contrast refractility, indicating that cell lysis had not occurred in these cells. This conclusion was supported by the direct fluorescence assays which showed >50% of the total fluorescence could be found in the medium of the infected culture under these conditions (following sonication), compared to ~5% for the uninfected culture (Fig. 6B).

**Figure 7:**
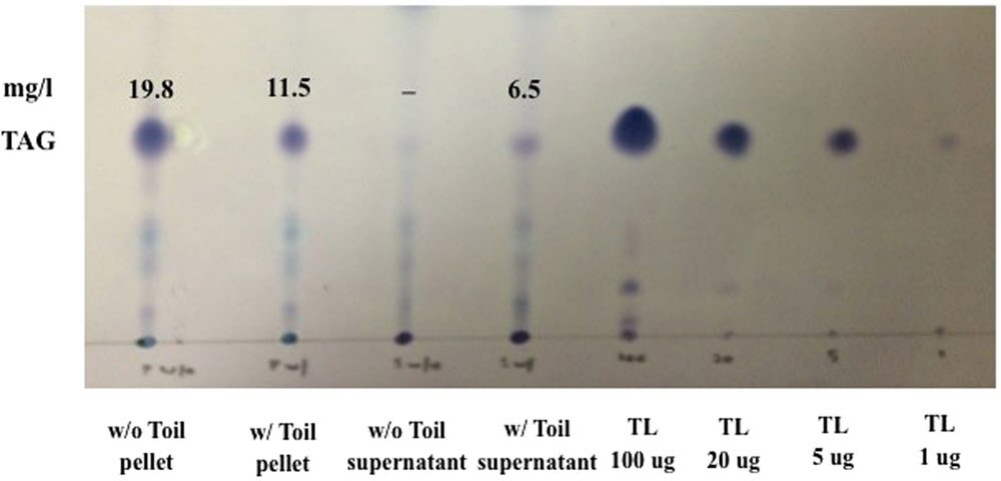
TLC analysis of lipid distribution. Samples were prepared from PD631 cells infected with phage Toil, as described in Methods. TAG was extracted from both supernatant and cell pellet fractions with chloroform/methanol mixture (2: 1, vol: vol),analyzed on a TLC plate. TAG mass calculated using a Glyceryl trioleate (TL) standard.

To quantify the localization of TAGs after phage infection, PD631 was grown under conditions for hyper-accumulation of lipid and then infected with Toil, as described in Methods. After 24-hour incubation, the cellular mass was separated by centrifugation and both cell and supernatant fractions subjected to chloroform/methanol (2:1, v/v), extraction and concentration in hexane. TLC analysis of these extracts revealed that ~ 30% of the total TAG was released to the supernatant portion in the infected culture (Fig. 7). The low TAG recovery in supernatant can be explained by attached TAGs on the cell membranes of lysed cells or trapped TAG granules which have a boundary layer with the cell debris.

The cell lysis results indicate that a biological, phage-mediated release method is a promising approach for the release of intracellular TAG. Future efforts will be focused on optimizing the infection conditions, assessing how much of the intracellular TAG is associated with cellular debris and, ultimately, engineering the phage itself for improved infection parameters. Nevertheless, even these initial results suggest that the bacteriophage approach will have considerable merit with respect to the issues of costs, safety, and environmental concerns, especially in comparison to traditional solvent extraction methods.

In conclusion, phage Toil exhibits no direct sequence similarity with other known tectiviral genomes at either the DNA or protein level, which marks it as the founder of a new species of *Tectivirus*. This phage was able to infect and lyse PD631 cells that had been induced for lipid accumulation. Moreover, these infections resulted in quantitative cell lysis and release of substantial fractions of soluble intracellular contents as well as ~30% of the intracellular TAG. Phage Toil thus represents a new tool for development of an economical and “bio-friendly” method for TAG extraction from *R. opacus*.

## Methods

### Bacterial strains and phage isolation

*R. opacus* PD630 (DSM 44193) was purchased from DSMZ, Germany. The strain was streaked on Reasoner’s 2 A (R2A) agar (Difco) plates for short-term (2 to 3 weeks) preservation. *R. opacus* PD631 is a domesticated derivative strain of PD630 achieved by subculturing PD630 on R2A agar for 10–12 transfers over the course of six months. Strain PD631 was routinely cultured at 30 °C with aeration using R2A broth medium.

To accumulate lipid to high levels, PD631 was grown in ammonium mineral salts medium (AMS)^34^ with 10 g/L gluconate to early stationary phase (OD_600_~10), followed by a second dose of 10 g/l gluconate to stimulate TAG accumulation. One liter of AMS contained 0.5 g of (NH_4_)Cl, 8.66 g of Na_2_HPO_4_, 1.71 g of K_2_SO_4_, 0.37 g of MgSO_4_ · 7H_2_O, 0.12 g of CaSO_4_ · 2H_2_O, 0.22 g of FeSO_4_ · 7H_2_O, 0.02 g of KI, 0.06 g of ZnSO_4_ · 7H_2_O, 0.03 g of MnSO_4_, 0.01 g of H_3_BO_3_, and 0.11 g of CoSO_4_.When cells reached early stationary phase, the culture was pelleted by centrifugation and the supernatant was discarded to remove the remaining NH_4_Cl in the medium. The cell pellet was resuspended in fresh nitrogen-free mineral salts medium (MS) with 10 g/l gluconate. To minimize utilization of the TAGs in the cells during stationary stage, no mixing was used to reduce oxygen supply during the TAG accumulation period. The culture vessel was sealed with a screw cap and incubated at 30 °C without shaking for 24 hrs (OD_600_~15).

### Phage isolation

Phage Toil was isolated by enrichment from a soil sample collected collected in Carter Creek wastewater treatment plant, College Station, TX. Fifty grams of soil was added to 100 mL of R2A broth in a 250 mL flask and agitated at 30 °C for 1 hour. The sample slurry was centrifuged at 10, 000 × g, 10 min, 4 °C to clear cells, and the supernatant was sterilized by passage through a 0.22 µm syringe-driven filter. Enrichment conditions were obtained by mixing 50 mL of the sterile filtrate with 0.5 mL of a fresh culture of PD631 and supplementing the mixture with, CaCl_2_ to 5 mM final concentration. After 24 hrs incubation at 30 °C, the enrichment mixture was cleared of cells by centrifugation and filter-sterilized. The filtrate was plated on 0.5% soft agar lawns of PD631. Individual plaques were picked and directly propagated on PD631 lawn using the confluent plate lysis method^39^.

### Adsorption tests

Adsorption tests were performed at 30 °C with shaking at 175 rpm. The *R. opacus* host strain was cultured in R2A to OD600~1.0. Phage was then added to at a multiplicity of infection (MOI)~0.004. Samples were taken at time intervals and diluted 100-fold in R2A medium precooled on ice. The resulting dilutions were centrifuged at 13, 000 × *g* for 2 min and the supernatant was titered as plaque forming units (PFU) per ml^40^.

### One-step growth analysis

*R. opacus* PD631 was cultured in R2A broth medium with 5 mM Ca^2+^ to OD600~0.5, pelleted at 10, 000 × g for 10 min and resuspended to a final OD600~10 in the same medium. Phage Toil was then added to the resuspended cells at input MOI~0.001. The mixture was incubated at 30 °C while shaking at 175 rpm for 10 min as preadsorption. Samples were then centrifuged at 5000 × g for 2 min and resuspended in fresh R2A medium with 5 mM Ca^2+^. The resuspension (30 μL) was diluted by 1000 × with fresh R2A media (30 mL) and shaken at 30 °C, 150 rpm. At each 30 min time interval, 100 μl of the infected culture was mixed with 100 μL *R. opacus* PD631, diluted appropriately and plated^39^.

### Transmission electron microscopy

Phage were prepared for microscopy by the Valentine method^41^ and stained with 2% (w/v) uranyl acetate. Grids were viewed in a JEOL 1200 EX transmission electron microscope under 100 kV accelerating voltage.

### Phase-contrast microscopy

To observe bacterial morphology during phage infection, a 5 μL sample of the infected culture was placed on a glass slide and then covered with a coverslip. Cells were imaged using an EC Plan-Neofluor 100 × objective installed on a Zeiss Axio Observer A1 microscope equipped an Axiocam HSM camera. All images were edited using the AxioVision software package (Zeiss).

### Fluorescence microscopy

Analysis was performed with a Nikon Ti-E microscope equipped with a CFI Plan Apo lambda DM 100 × objective, a Prior Scientific Lumen 200 illumination system, a UV-2E/C DAPI (4′,6-diamidino-2-phenylindole) filter cube, and a CoolSNAP HQ2 monochrome camera. All images were captured with NIS Elements Advanced Research software (version 4.10) and processed with ImageJ software (National Institutes of Health, Bethesda, MD).

### Fluorescence reporter

A PCR fragment containg the mCherry coding sequence was generated with the primers mC_SalI_F (5′-GTCGACGGTACCGTCAGAGAGATTGTTG-3′) and mC_EcoRI_R (5′-GAATTCGGATCCGGTTTACTTGTACAGC-3′) using pRE-mCherry as template. The PCR product was digested with SalI and EcorI and inserted into the cognate cloning sites of pTACHis18 (Xiong 2002). The plasmid pTACHis18-mCherry was electroporated into PD631 competent cells (Kalscheuer, 1999). Electroporation was performed using a Bio-Rad micropulser in electrocuvettes with gaps of 2 mm and with the following settings: ec2: 2.5 kV, 600 ohm, 25 uF. To quantify the fluorescence of mCherry-containing samples, 50 μl samples were measured in polystyrene 96-well plates (Corning) in a Tecan Infinite M200 Pro microplate reader.

### Lipid extraction and thin layer chromatography (TLC)

A 13 mL aliquot of bacterial culture was pelleted, resuspended in DI water and extracted with 10 mL of chloroform/methanol mixture (2: 1, vol: vol) as described^13^. A 5 mL volume from the (bottom) chloroform layer was transferred to a new glass vial, dried with an air stream. The dried lipid was resuspended in 100 μL of hexane.

Ten microliter (μL) of lipid samples (in hexane) and glyceryl trioleate (TL) standards (ranging from 1 to 100 μg) were applied to silica gel TLC plates (Product No. 4850–820, Whatman, Piscataway, NJ) and separated in a solvent system of hexane: diethyl ether: acetic acid (80: 20: 1, vol: vol)^5^. After separation, the TLC plate was dried, rinsed with 1 M sodium chloride solution, stained in a 0.2% (wt/vol in 1 M sodium chloride) amido black solution followed by color development as described before^13^.

The TAG content of each sample was determined by analyzing the image of the TLC plate using ImageJ software (National Institutes of Health, Bethesda, MD). A TAG standard curve was developed by correlating the loaded amount (μg) of TAG standards to “(area)/(mean gray value)” of the relevant spots developed on the TLC plate. The standard curve was then used to determine the TAG content of the each sample based on the corresponding spot on the TLC plate.

### Phage DNA sequencing and annotation

Phage genomic DNA was extracted from 10 mL of high-titer plate lysate using a modified Promega Wizard kit method as described previously^42^. Phage DNA was prepared for sequencing using an Illumina Truseq Nano V2500 bp library kit and sequenced in the Illumina MiSeq platform. Sequence reads were quality controlled by FastQC (www.bioinformatics.babraham.ac.uk/projects/fastqc) and trimmed with FastX Toolkit (http://hannonlab.cshl.edu/fastx_toolkit) before assembly in SPAdes v3.5.0^43^. Structural annotation was conducted using Glimmer3^44^ and MetaGeneAnnotator^45^ and gene functions predicted by InterProScan^46^, TMHMM (http://www.cbs.dtu.dk/services/TMHMM), BLAST^47^ and HHpred^48^. The annotated phage genome was deposited in NCBI Genbank under accession no. KY817360.

### Ethics approval and informed consent

Not applicable.

### Data Availability Statement

All data generated or analysed during this study are included in this published article and its supplementary information files.

## Electronic supplementary material

Supplementary information
