# Supplementary material for: Characterization of a Novel Tectivirus Phage Toil and Its Potential as an Agent for Biolipid Extraction
Source: Sci Rep. 2018 Jan 18;8:1062. doi: 10.1038/s41598-018-19455-2 (PMC5773508; doi:10.1038/s41598-018-19455-2)
Supplement: Supplementary file 1 — Supplementary information [file 41598_2018_19455_MOESM1_ESM.pdf]

Supplementary Information

for

Characterization of a Novel *Tectivirus* Phage Toil and Its Potential as an Agent for Biolipid Extraction

Jason J. Gill[1,3]; Baixin Wang[2]; Emily Sestak[2]; Ryland Young[3,4]; and Kung-Hui Chu[1*]

[1]Department of Animal Science, [2]Zachry Department of Civil Engineering, [3]Center for Phage Technology, [4]Department of Biochemistry and Biophysics
Texas A&M University, College Station, TX 77843

*Corresponding author

Mailing address: 402A DLEB, Zachry Department of Civil Engineering, Texas A&M University, 3136 TAMU, College Station, TX 77843, USA

E-mail: kchu@civil.tamu.edu; Phone: +1- (979) 845-1403; Fax: +1- (979) 862-1542

Supporting Information: 5 pages total, 3 figures and 1 table.

Figure S1. Minimal Inhibition Concentration (MIC) tests. *Rhodococcus opacus* PD631 were grown in R2A broth with 5 mM $CaCl_2$ to exponential growth phase, and diluted to 10^4 CFU/ml, 5 ml aliquots were transferred to culture tubes. Phage Toil lysate was diluted in series, and applied to each culture tube. The input multiplicity of infection (MOI) was 0, 0.01, 0.1, 1, 10 or 100. All tubes was incubated at 30 °C for 30 hrs.

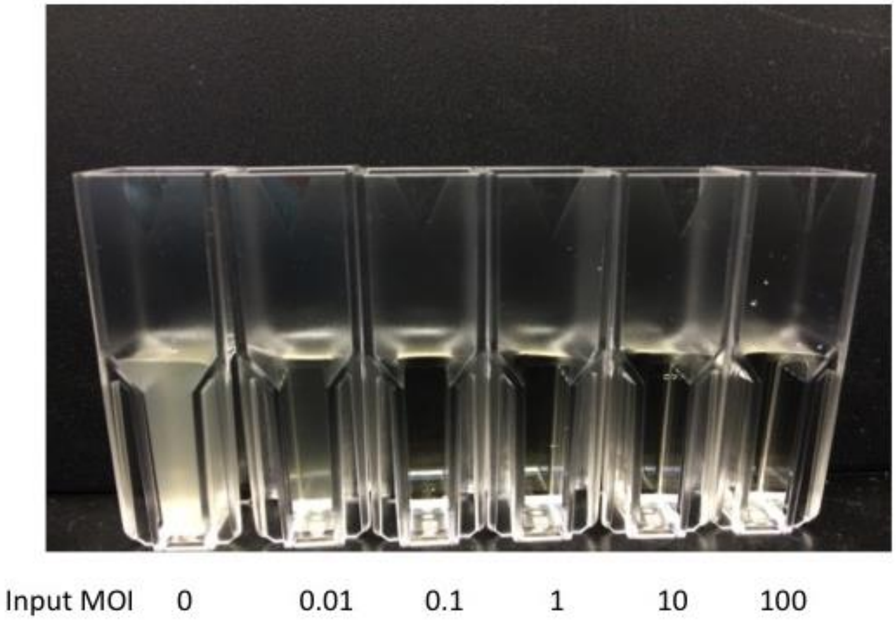

Figure S2. Optimization of Ca^{2+}or Mg^{2+} concentrations for phage Toil adsorption. P10 is the free phage titer at 10 min in the supernatant, P0 is the free phage titer at time zero.

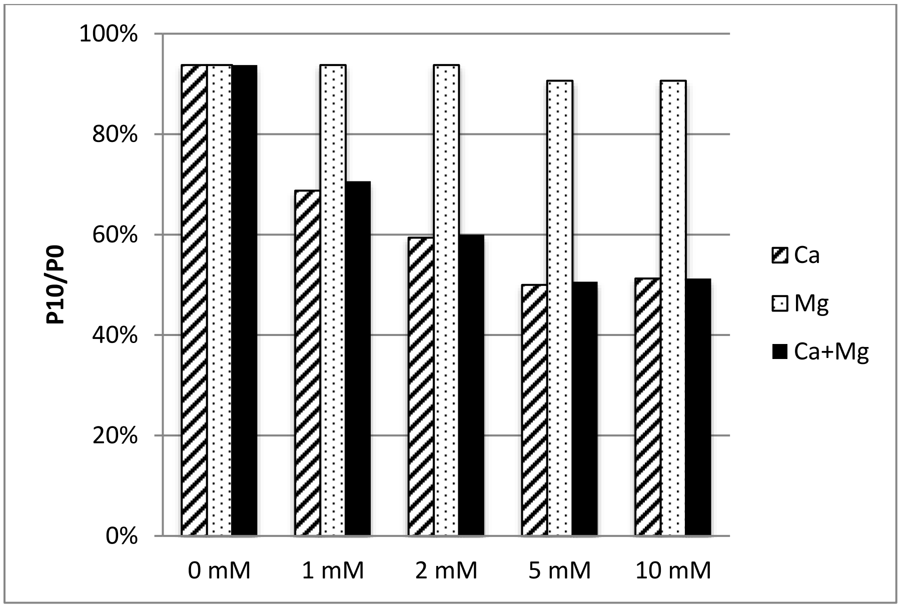

Figure S3. Restriction digest of phage Toil genomic DNA with and without proteinase K treatment. Lane 1, NEB 1 kb molecular weight standard; Line 2 Lambda HindIII fragments; Lane 3, Untreated DNA; Lane 4, proteinase K treated DNA; Lane 5, HindIII digested DNA; Lane 6, HindIII digested and proteinase treated; Line 7, HindIII cut and 60°C heated; Line 8, HindIII cut, 60°C heated and proteinase treated.

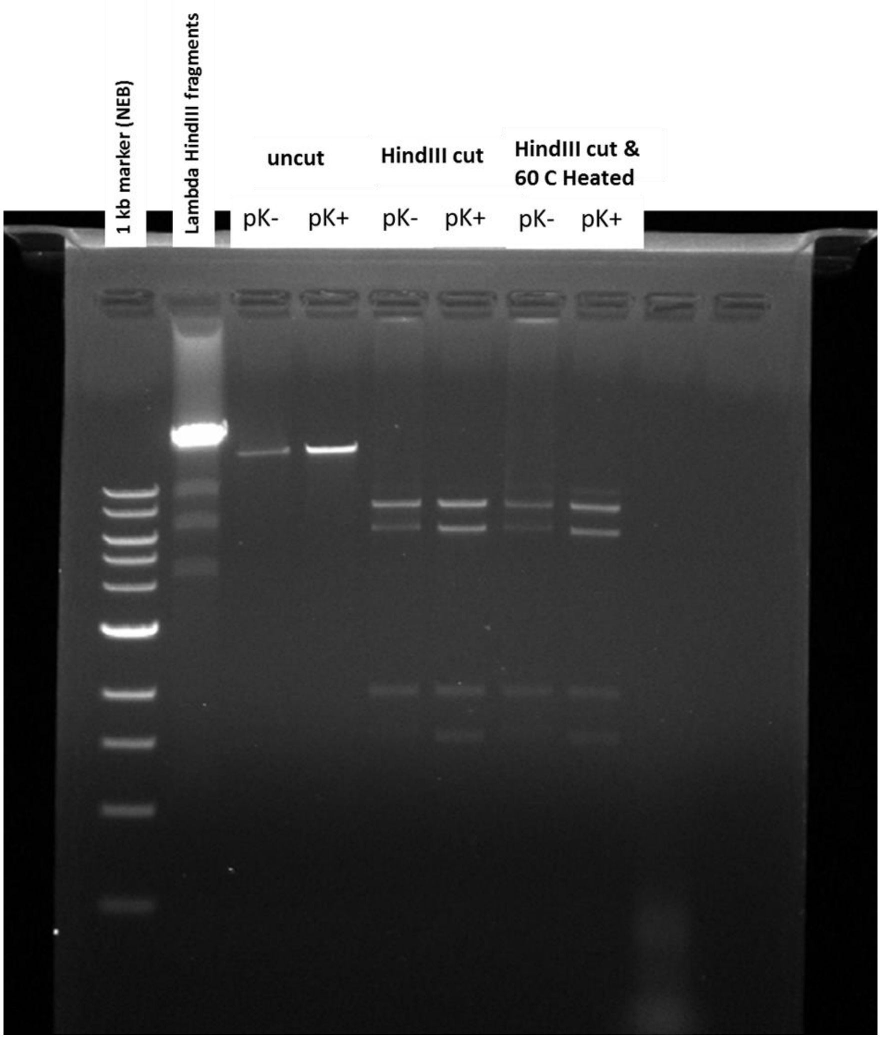

Table S1. Predicted genes and protein functions of bacteriophage Toil, a novel Tectivirus infecting *Rhodococcus opacus* (GenBank accession KY817360).

Gene	CDS coordinates	Strand	Predicted product	Conserved domains	NCBI Protein ID
1	90..359	-	hypothetical protein		ARK07684
2	356..559	-	hypothetical protein		ARK07685
3	617..892	-	hypothetical protein		ARK07686
4	1011..1247	-	hypothetical protein		ARK07687
5	1254..1679	-	hypothetical protein		ARK07688
6	1731..2018	-	hypothetical protein		ARK07689
7	2015..3805	-	DNA polymerase	IPR004868, IPR023211	ARK07690
8	3732..4181	-	hypothetical protein		ARK07691
9	4245..4781	-	hypothetical protein		ARK07692
10	4865..5299	+	hypothetical protein		ARK07693
11	5296..5793	+	hypothetical protein		ARK07694
12	5783..6499	+	DNA packaging ATPase	IPR027417	ARK07695
13	6503..6637	+	hypothetical protein		ARK07696
14	6654..7796	+	coat protein	HHpred: STIV coat 2bbd, 97.7%	ARK07697
15	7809..7925	+	hypothetical protein		ARK07698
16	7937..8059	+	hypothetical protein		ARK07699
17	8019..8381	+	hypothetical protein		ARK07700
18	8359..8508	+	hypothetical protein		ARK07701
19	8510..9184	+	hypothetical protein		ARK07702
20	9181..10167	+	LysM domain protein	IPR018392, IPR036779	ARK07703
21	10177..10395	+	hypothetical protein		ARK07704
22	10419..10586	+	hypothetical protein		ARK07705
23	10595..11254	+	entry lysozyme	IPR023346	ARK07706
24	11251..11715	+	hypothetical protein		ARK07707
25	11712..12053	+	hypothetical protein		ARK07708
26	12053..12712	+	CHAP-domain endopeptidase	IPR000064	ARK07709
27	12705..12992	+	hypothetical protein		ARK07710
28	12992..13621	+	hypothetical protein		ARK07711
29	13618..14049	+	hypothetical protein		ARK07712
30	14049..15284	+	receptor-binding protein	IPR008983	ARK07713
31	15291..15974	+	LysA-like endolysin	IPR009045	ARK07714
32	16001..16222	+	hypothetical protein		ARK07715
33	16327..16605	+	hypothetical protein		ARK07716
34	16602..16784	+	hypothetical protein		ARK07717
35	16874..17161	+	hypothetical protein		ARK07718
